# Predicting protein functions using incomplete hierarchical labels

**DOI:** 10.1186/s12859-014-0430-y

**Published:** 2015-01-16

**Authors:** Guoxian Yu, Hailong Zhu, Carlotta Domeniconi

**Affiliations:** 1 Department of Computer Science, Hong Kong Baptist University, Kowloon Tong, Hong Kong China; 2 College of Computer and Information Sciences, Southwest University, Chongqing, China; 3 Department of Computer Science, George Mason University, Fairfax, VA USA

**Keywords:** Function prediction, Incomplete hierarchical labels, Combined similarity, Gene ontology

## Abstract

**Background:**

Protein function prediction is to assign biological or biochemical functions to proteins, and it is a challenging computational problem characterized by several factors: (1) the number of function labels (annotations) is large; (2) a protein may be associated with multiple labels; (3) the function labels are structured in a hierarchy; and (4) the labels are incomplete. Current predictive models often assume that the labels of the labeled proteins are complete, i.e. no label is missing. But in real scenarios, we may be aware of only some hierarchical labels of a protein, and we may not know whether additional ones are actually present. The scenario of incomplete hierarchical labels, a challenging and practical problem, is seldom studied in protein function prediction.

**Results:**

In this paper, we propose an algorithm to *P*redict protein functions using *I*ncomplete hierarchical *L*abe*L*s (PILL in short). PILL takes into account the hierarchical and the flat taxonomy similarity between function labels, and defines a *Com*bined *Sim*ilarity (*ComSim*) to measure the correlation between labels. PILL estimates the missing labels for a protein based on *ComSim* and the known labels of the protein, and uses a regularization to exploit the interactions between proteins for function prediction. PILL is shown to outperform other related techniques in replenishing the missing labels and in predicting the functions of completely unlabeled proteins on publicly available PPI datasets annotated with MIPS Functional Catalogue and Gene Ontology labels.

**Conclusion:**

The empirical study shows that it is important to consider the incomplete annotation for protein function prediction. The proposed method (PILL) can serve as a valuable tool for protein function prediction using incomplete labels. The Matlab code of PILL is available upon request.

**Electronic supplementary material:**

The online version of this article (doi:10.1186/s12859-014-0430-y) contains supplementary material, which is available to authorized users.

## Background

The increasing amount of proteomic data produced using high-throughput technology makes it crucial but challenging to develop computational models that can identify hypothetical functions of proteins. Such techniques have the potential to drive the biological validation and discovery of novel functions of proteins, and to save on the experimental cost. At the same time, functional annotations of proteins have been incorporated into several bioinformatics tools (e.g., Panther [1], IntPath [2], and InterProScan [3]) to investigate the semantic similarity between proteins, proteins functional interactions, pathway enrichment analysis, functional enrichment analysis, and phylogenetic tree [4,5].

Protein function prediction is a challenging computational problem, characterized by several intrinsic hardships: the number of function labels is rather large, each protein can have several labels, and the labels are structured in a hierarchy and are unbalanced. Furthermore, function labels associated to proteins are uncertain and incomplete. Various computational models have been proposed to address one or more of these issues [3,6-10]. Some models use cost-sensitive learning and hierarchical classification [8,11], others apply multi-label learning [12,13], classifier ensemble [8,12] and multiple networks (kernel) integration [14] to use the complimentary information spread across different heterogeneous data sources. More recent approaches incorporate evolutionary knowledge [15], pathways [1,2,16], domains [17], or negative examples selection [7,18]. For a complete review on protein function prediction, see [6,10,19]. Radivojac *et al.* [9,19] organized the large scale community-based critical assessment of protein function annotation, and suggested that there is significant room for improving protein function prediction.

Protein function prediction can be viewed as a multi-label learning problem [7,10,12,20,21]. Recently, multi-label learning approaches that use the correlation (or similarity) between function labels have been introduced. Pandey *et al.* [22] incorporated label correlation using Lin’s similarity [23] into the *k*-nearest neighborhood (LkNN) classifier; the authors observed that utilizing the correlation between function labels can boost the prediction accuracy. Zhang and Dai [24] investigated the usefulness of functional interrelationships based on Jaccard coefficients for protein function prediction. Wang *et al.* [25] introduced a function-function correlated multi-label learning method to infer protein functions. Yu *et al.* [12] studied a directed bi-relational graph (composed by protein nodes and function label nodes) to utilize the correlation between function labels for protein function prediction. Chi and Hou [26] assumed the label sets of two proteins can influence their similarity and introduced a Cosine Iterative Algorithm (CIA). In each iteration of CIA, the function predicted with highest confidence is appended to the label set of a protein. Next, the pairwise similarity between training proteins and testing proteins is updated based on the extended function sets. CIA considers the updated pairwise similarity, the function correlation based on cosine similarity, and the PPI network topology to predict functions in consecutive iterations.

Most of these multi-label learning algorithms focus on exploiting label correlations to boost prediction accuracy, under the assumption that the labels of labeled proteins used for training are complete, i.e. no label is missing. Due to various reasons (e.g., evolving Gene Ontology terms, or limitations of experimental methods), in practice we may be aware of some functions only, while additional functions (unknown to us) may also be associated with the protein. In other words, proteins are partially labeled. Learning from partially and multi-label instances (or proteins) can be formulated as a multi-label and weak-label learning problem [27-29].

Several multi-label and weak-label learning algorithms have been introduced in the past years. Sun *et al.* [27] studied a multi-label and weak-label learning method called WELL. WELL assumes there is a margin between instances of different classes and any given label has a small number of member instances. To make use of the label correlation among multi-label instances, this approach assumes that there is a group of low rank based similarities, and the similarity between instances of different labels can be approximated based on these similarities. However, WELL relies on quadratic programming to compute the low rank based similarities and to make the final predictions. Therefore, it’s computationally expensive and can hardly make predictions for samples with a large number of labels. Bucak *et al.* [30] proposed a weak-label learning approach called MLR-GL. MLR-GL optimizes a convex objective function that includes a ranking loss and a group Lasso loss. MLR-GL aims at labeling instances with no labels by using partially labeled instances. Yang *et al.* [28] introduced a multi-instance and multi-label weak-label learning algorithm. Yu *et al.* [29] proposed an approach called ProWL to predict protein functions using partially labeled proteins. ProWL exploits the label correlation and available labels of a protein to estimate the likelihood of a missing function for the protein. ProWL integrates these estimations with a smoothness loss function to replenish the missing function labels and to predict functions for proteins with no labels. Yu *et al.* [31] assumed a function label depends on the feature information of proteins and introduced an algorithm called ProDM. ProDM maximizes this dependency to replenish the missing function labels and to predict functions for unlabeled proteins.

However, these weak-label learning techniques only use the *flat* relationships among function labels, and do not explicitly take into account the *hierarchical* relationship among labels. It is widely recognized that the MIPS Functional Catalogue (FunCat) [32] organizes the function labels in a tree structure and the Gene Ontology (GO) [33] organizes the function terms (or labels) in a directed acyclic graph. It is reported that exploiting the hierarchical relationship among function labels can boost the accuracy of protein function prediction [7,8,11,22]. For example, Barutcuoglu *et al.* [11] suggested that organizing the prediction produced by the binary classifier for each individual function label in a Bayes network can improve the accuracy of gene function prediction. Tao *et al.* [34] utilized an information theory based metric to measure the interrelationships between function labels and to determine whether a certain function label belongs to a protein or not. However, this method cannot predict functions for unlabeled proteins, since it only employs the known annotations of a protein to infer its other potential annotations. Jiang *et al.* [35] combined the relational PPI network and the label hierarchical structure to predict consistent functions by setting the descendants of a function label as negative whenever this label is set to negative. Pandey *et al.* [22] used Lin’s similarity to capture the relationship among hierarchically organized labels. Schietgat *et al.* [36] integrated hierarchical multi-label decision trees for protein function prediction. Valentini [7] post-processed the prediction made by a binary classifier for each label according to the true path rule in the GO and the FunCat hierarchies, and proposed a method called TPR. Cesa-Bianch *et al.* [8] integrated cost-sensitive learning and data fusion with TPR to further boost the accuracy of protein function prediction. Valentini [10] advocated in his recent survey that it is paramount to exploit the hierarchical relationship among function labels for protein function prediction.

According to the *True Path Rule* [7] in GO and FunCat: (i) if a protein is labeled with a function, then this protein should be labeled with the ancestor functions (if any) of this function; (ii) if a protein cannot be labeled with a function, then this protein should not be labeled with the descendant functions (if any) of this function. In [29,31], the incomplete annotation problem was simulated by randomly masking function labels in a flat style, ignoring the hierarchical relationship between labels. In the simulation, if a function label of a protein is missing, this protein may still be labeled with the descendant functions of this function. And in fact, the missing function can be directly inferred from its descendant function labels.

In this paper, we studied the incomplete label problem in a hierarchical manner, as opposed to a flat style. We propose an approach called PILL to predict protein functions using partially labeled proteins with hierarchical labels. PILL integrates the hierarchical and flat relationships between function labels to estimate the likelihoods of missing labels, and the interaction between proteins to replenish the missing annotations and to predict the functions of unlabeled proteins. Particularly, PILL simulates the incomplete hierarchical labels by randomly masking the leaf function labels of a protein, which is closer to the real situation than the simulation in the previous study [29,31]. We conducted experiments on three publicly available PPI datasets, in which each dataset was annotated with FunCat labels and GO labels. The experimental results showed that PILL outperforms other related algorithms on replenishing the missing labels of partially labeled proteins and on predicting functions for completely unlabeled proteins.

## The incomplete hierarchical label problem

**Figure 1:**
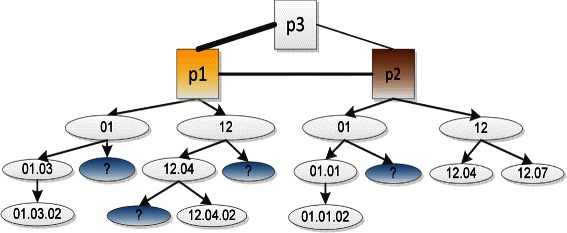
**Illustration of incomplete hierarchical labels for proteins annotated with MIPS FunCat labels.** A rectangle represents a protein (*pi*, *i*∈{1,2,3}); an ellipse denotes a function label, and a undirected line between rectangles captures a protein-protein interaction (the more reliable the interaction is, the thicker the line is). All the functional labels (including the missing function labels denoted by color ellipses with question marks ‘?’) in the ellipses should be associated with the proteins, but only the functional labels in the white ellipses are known. For better visualization, other functional labels (i.e., ‘12.03’ and ‘03’, which are not ground-truth labels for these proteins), are not plotted in the Figure.

Figure 1 illustrates an example of an incomplete hierarchical label problem for proteins annotated with FunCat labels. A corresponding example for the GO labels is given in Figure S1 of the Additional file 1. In Figure 1, *p*1 and *p*2 are partially labeled (missing labels are described by a question mark ?), and *p*3 is completely unlabeled. Note, other FunCat labels (i.e., ‘12.03’ and ‘03’) are not really missing for these proteins, and thus not shown in the figure; these function labels will also be viewed as candidate ‘missing’ labels. The missing labels are leaf function labels. If a non-leaf function label of a protein is missing, we can directly append this function label to this protein from its descendant function labels. Each hierarchy of non-leaf and leaf function labels is defined with respect to a single protein. For example, ‘12.04’ is a leaf function label for *p*2, but it is a non-leaf function label for *p*1, since *p*1 is labeled with a descendant label (‘12.04.02’) of ‘12.04’. Our task is to replenish the missing labels of *p*1 and *p*2, and to predict functions for *p*3. To this end, we define three kinds of relationships between function labels: (i) parent-child (e.g., ‘01.03’ is a child function label of ‘01’); (ii) grandparent-grandson (e.g., ‘01.03.02’ is a grandson label of ‘01’); and (iii) uncle-nephew (e.g., if we consider ‘01’ as a sibling of ‘12’, although these two labels do not have an explicit common parent label, ‘12’ is an uncle label of ‘01.03’). These relationships will be further discussed in the next Section.

## Methods

### Function correlation definition

A protein often has multiple functions, which are organized as a tree hierarchy (FunCat) or as a directed acyclic graph (GO). Some pioneers [7,10,11,22] have demonstrated that exploiting the hierarchical relationship among function labels can boost the performance of protein function prediction. Pandey *et al.* [22] used the Lin’s similarity [23] to take advantage of the hierarchical relationship between function labels. Lin’s similarity measures the similarity of two function labels in terms of their proximity in the hierarchical ontology, as well as their content. It is defined as follows:

(1) $$ LinSim(s,t)=\frac{2 \times \log p_{ca}(s, t)}{\log p(s)+ \log p(t)},   $$

and

(2) $$ p_{ca}(s, t) = \min_{k \in ca(s, t)} p(k)   $$

*s* and *t* are two function labels, *p*(*s*) denotes the probability for a protein to be labeled with *s*. *p*(*s*) can be estimated from the available number of member proteins of *s* for an organism. *c* *a*(*s*,*t*) is the set of *common ancestors* of *s* and *t*, and *p*_*ca*_(*s*,*t*) denotes the probability of the most specific function label in the hierarchy that subsumes both *s* and *t*. Intuitively, Eq. (1) measures the semantic similarity of *s* and *t* in terms of the content of their minimum subsumer node in the hierarchy. Clearly, *p*_*ca*_(*s*,*t*)=1 if *s*=*t*, and *p*_*ca*_(*s*,*t*)=0 when their minimum subsumer is the root node of the ontology, or the function label corresponding to the minimum subsumer node is associated with all the proteins of an organism. *L* *i* *n* *S* *i* *m*(*s*,*t*) can also be viewed as a correlation measure between *s* and *t*. According to this definition, *L* *i* *n* *S* *i* *m*(*s*,*t*) is large if *s* and *t* often co-annotate the same proteins, and their most specific ancestor label is close to *s* and *t* but far away from the root node. On the other hand, if the most specific ancestor of *s* and *t* is (close to) the root node, but *s* and *t* are far away from the root node in the hierarchy, *L* *i* *n* *S* *i* *m*(*s*,*t*) will be small.

However, if *s* is an ancestor of *t*, taking *s* as the common ancestor of *t* is preferable to any other common ancestor label, since *s* is more specific than any other label in the common ancestor label set, and *s* also subsumes both *s* and *t*. The more specific the function, the fewer member proteins this function has, and the smaller the probability is for a protein to be labeled with this function. Therefore, we substitute *p*_*ca*_(*s*,*t*) with *p*_*sa*_(*s*,*t*), which is defined as follows:

(3) $$ p_{sa}(s, t) = \min_{k \in sa(s, t)} p(k)   $$

*s* *a*(*s*,*t*) represents the set of *shared ancestors* of *s* and *t*, which includes *s* if *t* is a descendant label of *s*, or *t* if *t* is an ancestor label of *s*. Thus, *c* *a*(*s*,*t*)⊆*s* *a*(*s*,*t*). We extend Lin’s similarity to a similarity named *H* *S* *i* *m*(*s*,*t*) by substituting *p*_*ca*_(*s*,*t*) in Eq. (1) with *p*_*sa*_(*s*,*t*). If *s* is an ancestor label of *t*, *H* *S* *i* *m*(*s*,*t*) is no smaller than *L* *i* *n* *S* *i* *m*(*s*,*t*), since *s* is more specific than any function label in *c* *a*(*s*,*t*) (or *p*(*s*)≤*p*_*ca*_(*s*,*t*)). When *s* and *t* are siblings (or cousins), *H* *S* *i* *m*(*s*,*t*) and *L* *i* *n* *S* *i* *m*(*s*,*t*) are the same.

*s* *a*(*s*,*t*) often includes more specific functions (i.e., the parent function label of *t*) than *c* *a*(*s*,*t*), since *c* *a*(*s*,*t*)⊆*s* *a*(*s*,*t*). If *t* is missing for a protein, but the ancestor function labels (including parent function label *s*) of *t* are associated with this protein, it is easy to see that the missing label estimation from the parent function is more reliable than that from other ancestor functions (i.e. grandparent functions). This property of function label hierarchies motivates us to estimate the missing labels using *HSim* instead of *LinSim*. The statistics computed in the next Section supports our rationale.

Nevertheless, when *s* and *t* have no shared ancestor (e.g., the function label in the first level of MIPS FunCat does not have an ancestor label), *p*_*sa*_(*s*,*t*)=0; when the most specific shared function label is associated with almost all the proteins (e.g., the function label corresponds to the root node of the GO biological process sub-ontology hierarchy), *p*_*sa*_(*s*,*t*)≈1 and *H* *S* *i* *m*(*s*,*t*)≈0. But *H* *S* *i* *m*(*s*,*t*)≈0 does not mean that *s* and *t* have no correlation. For example, there are 272 proteins in *S. Cerevisiae* labeled with ‘40’ (CELL FATE), 448 proteins labeled with ‘43’ (CELL TYPE DIFFERENTIATION), and 170 proteins labeled with both ‘40’ and ‘43’. If a protein is labeled with ‘40’ and it is unknown whether this protein has ‘43’, we have 170/272=62.5*%* confidence that this protein is also labeled with ‘43’. However, neither *HSim* nor *LinSim* can provide this confidence. The reason is that ‘40’ and ‘43’ do not have any shared ancestor label, and both of them only consider the *hierarchical* relationship between function labels. In fact, it is observed that *flat* label relationships are also beneficial for protein function prediction [24,25,29]. To overcome this limitation of *H* *S* *i* *m*(*s*,*t*), we introduce a *C* *o* *m* *S* *i* *m*(*s*,*t*) to describe the correlation between function labels:

(4) $$ ComSim(s, t)= \left\{ \begin{array}{l} HSim(s, t), \ \text{if} \ p_{sa}(s,t)\in (0, 1) \\ JcdSim(s, t), \ \text{otherwise} \\ \end{array} \right.   $$

where *JcdSim* is the similarity based on the Jaccard coefficient *J* *c* *d* *S* *i* *m*(*s*,*t*)=|*N*(*s*)∩*N*(*t*)|/|*N*(*s*)∪*N*(*t*)|. *N*(·) denotes the set of proteins labeled with the corresponding function label and |*N*(·)| is the cardinality of the set. From the definition, if *s* and *t* do not have shared ancestor function labels, *C* *o* *m* *S* *i* *m*(*s*,*t*) is large when they often co-associated with the same set of proteins; *C* *o* *m* *S* *i* *m*(*s*,*t*) is small when they seldom co-associated with the same proteins. When *s*, *t* and the most specific shared ancestors of these two function labels are always associated with the same proteins, *C* *o* *m* *S* *i* *m*(*s*,*t*)=1. In this case, *J* *c* *d* *S* *i* *m*(*s*,*t*) is also set to 1. As such, *ComSim* captures both the hierarchical and the flat relationships between functions.

### Statistics of hierarchical function label relationships

From the true path rule of function label hierarchies, it’s easy to observe that:

- *p*(*s*|*p* *a* *r*(*s*))≥*p*(*s*|*g* *p* *a* *r*(*s*))
- *p*(*s*|*g* *p* *a* *r*(*s*))≥*p*(*s*|*u* *n* *c* *l* *e*(*s*))

where *p* *a* *r*(*s*) denotes the parent function label of *s*, *g* *p* *a* *r*(*s*) is the grandparent function label of *s*, and *u* *n* *c* *l* *e*(*s*) is the uncle (parent’s sibling) function label of *s*. *p*(*s*|*p* *a* *r*(*s*)) is the conditional probability that a protein is labeled with *s* given that it’s already labeled with *p* *a* *r*(*s*). These equations hold since if a protein is labeled with *s*, then this protein is also labeled with the ancestor functions of *s* (including *p* *a* *r*(*s*) and *g* *p* *a* *r*(*s*)), and if a protein is labeled with *u* *n* *c* *l* *e*(*s*), this protein is also labeled with *g* *p* *a* *r*(*s*). In contrast, if a protein is labeled with *p* *a* *r*(*s*) (or *g* *p* *a* *r*(*s*)), it is uncertain whether this protein is labeled with *s* (or *u* *n* *c* *l* *e*(*s*)).

**Figure 2:**
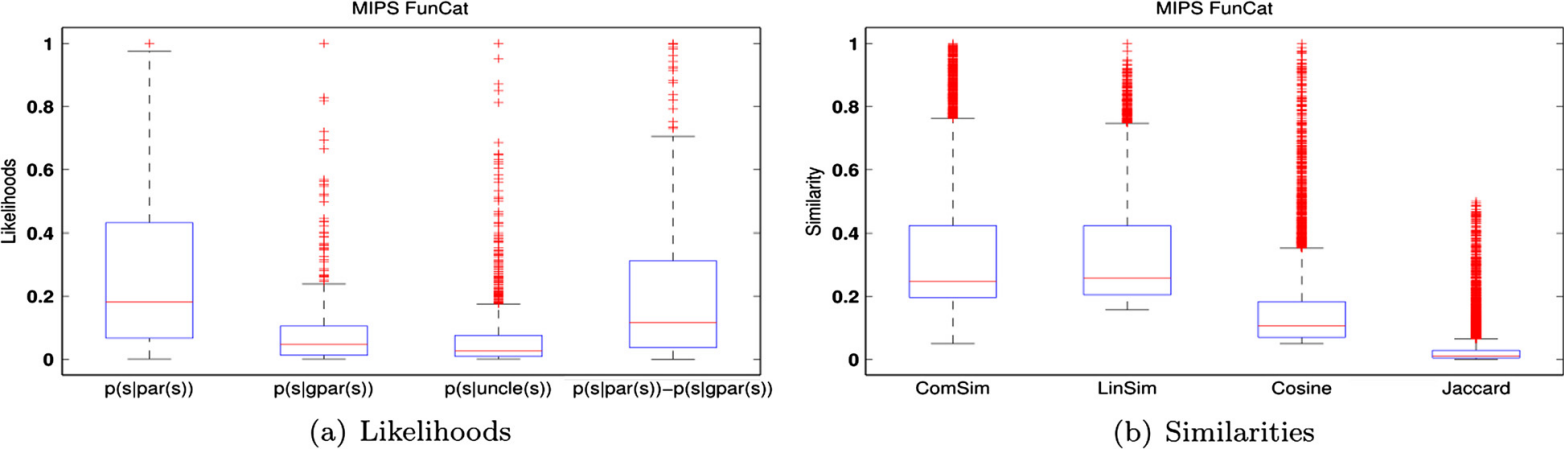
**Label relationship statistics and four label similarities on proteins of S. Cerevisiae annotated with FunCat labels.** In the figure, each boxplot describes the distribution of a likelihood (or similarity), the central line is the median, the edges of the box are the 25% and 75% percentiles, the whiskers extend to the most extreme datapoints that are not considered as outliers, and the outliers are plotted individually as ‘+’.

Based on these rules, we investigate the parent-child relationship by counting the cases in which a protein is labeled with both a function label in *p* *a* *r*(*s*) and with *s*. Similarly, we investigate the grandparent-grandson (or uncle-nephew) relationship by computing the cases in which a protein is labeled with both a label in *g* *p* *a* *r*(*s*) (or *u* *n* *c* *l* *e*(*s*)) and with *s*. The distributions of these three statistics for proteins in *S. Cerevisiae* (labeled with FunCat labels) are shown in the first three boxplots in Figure 2. In addition, we report *p*(*s*|*p* *a* *r*(*s*))−*p*(*s*|*g* *p* *a* *r*(*s*)) in the fourth boxplot in Figure 2. We also provide the distribution of all pairs of function correlations based on the proposed *ComSim*, Lin’s similarity, Cosine similarity, and Jaccard coefficients on the same protein data in Figure 2. The corresponding distributions obtained on the *S. Cerevisiae* proteins labeled with GO labels are given in Figure S2 of the Additional file 1. For a fair comparison, all the zero elements in these likelihoods and similarities are removed, since some pairwise function labels do not have the hierarchical (i.e., parent-child) relationships, or are not associated with the same proteins.

The boxplots of Figure 2 support the relationships *p*(*s*|*p* *a* *r*(*s*))≥*p*(*s*|*g* *p* *a* *r*(*s*)) and *p*(*s*|*g* *p* *a* *r*(*s*))≥*p*(*s*|*u* *n* *c* *l* *e*(*s*)). If *s* is missing for a protein, and the protein is labeled with labels in *p* *a* *r*(*s*), *g* *p* *a* *r*(*s*) and *u* *n* *c* *l* *e*(*s*), the estimated likelihood of the missing label *s* from *p* *a* *r*(*s*) is more reliable than that from *g* *p* *a* *r*(*s*) and *u* *n* *c* *l* *e*(*s*). The explanation is straightforward: the more specific the function label is, the fewer member proteins the label has. In other words, if the function label in *p* *a* *r*(*s*) is associated with a protein, we can ensure that the function label in *g* *p* *a* *r*(*s*) is also associated with the same protein, but not vice versa. Similarly, given that *u* *n* *c* *l* *e*(*s*) is the sibling of *p* *a* *r*(*s*) and the two share the same parent, if a protein is annotated with *u* *n* *c* *l* *e*(*s*), this protein is also annotated with *g* *p* *a* *r*(*s*). Similar results are obtained for the *S. Cerevisiae* proteins annotated with GO labels (see Figure S2 of the Additional file 1).

In Figure 2, *p*(*s*|*p* *a* *r*(*s*)) is more evenly distributed than *p*(*s*|*g* *p* *a* *r*(*s*)) and *p*(*s*|*u* *n* *c* *l* *e*(*s*)), and it has fewer outliers than the latter two. We can also observe that the distributions of the function correlations defined by *LinSim* and *ComSim* are closer to *p*(*s*|*p* *a* *r*(*s*)) than the correlations defined by the Cosine similarity and the Jaccard coefficient, and the label correlations based on *LinSim* and *ComSim* are more evenly distributed than the correlations based on Cosine and Jaccard similarity, since the former two have fewer outliers than the latter two. *ComSim* considers both the hierarchical (measured by *HSim*) and flat (measured by *JcdSim*) relationships among labels, and its margin between 25% and 75% percentiles is wider than that of *LinSim*. In addition, the overlap between *ComSim* and *p*(*s*|*p* *a* *r*(*s*)) is larger than that between *LinSim* and *p*(*s*|*p* *a* *r*(*s*)). In fact, we also studied the Gaussian function ($exp\left (-\frac {(x-\mu)^{2}}{\sigma ^{2}}\right)$, where *μ* and *σ* are the mean and standard deviation of *x*, *x* corresponds to a kind of likelihood or similarity) distribution of these likelihoods and similarities, and also observed that *ComSim* overlaps more with *p*(*s*|*p* *a* *r*(*s*)) than with other similarity metrics (not reported). Since *ComSim* will be used to estimate the likelihoods of missing labels, these differences indicate that *ComSim* can estimate the missing labels more accurately than the other three techniques. The advantage of *ComSim* will also be verified in our experiments.

### Objective function

Given *n* proteins, let *K* be the number of distinct functions across all proteins. Let *Y*=[**y**_1_,**y**_2_,…,**y**_*n*_] be the original label set, with *y*_*ik*_=1 if protein *i* has the *k*-th function, and *y*_*ik*_=0 if it’s unknown whether this protein has the *k*-th function or not. We assume the first *l*≤*n* proteins are partially labeled and the remaining *n*−*l* proteins are completely unlabeled. We set the normalized function correlation matrix as $C_{m}(s,t)=\frac {ComSim(s,t)}{\sum _{t=1}^{K} ComSim(s,t)}$.

Based on the definition of *C*_*m*_, we can estimate the likelihood of a missing function label on the *i*-th (*i*≤*l*) partially labeled protein as follows:

(5) $$ \tilde{y}_{ik}= \left\{ \begin{array}{l} \mathbf{y}^{T}_{i} C_{m}(\cdot, k), \text{if} \ y_{ik}=0\\ 1, \quad \ \text{otherwise}\\ \end{array} \right.   $$

If *y*_*ik*_=0 and the *k*-th function label has a large correlation with the already known functions of protein *i*, then it is likely that the *k*-th function is missing for this protein, $\tilde {y}_{\textit {ik}}$ is assigned to a large value. $\tilde {\mathbf {y}}_{i}$ is the label vector for the confirmed labels (the corresponding entries are set to 1) together with **y**_*i*_ and *C*_*m*_ estimated likelihoods of the missing labels (for entries corresponding to *y*_*ik*_=0) on the *i*-th protein.

Based on $\tilde {\mathbf {y}}_{i}$, we can define the empirical loss function on *l* partially labeled proteins as follows:

(6) $$ \begin{aligned} \Psi_{1}(f)&=\min_{f}\sum_{i=1}^{l}\left\|\mathbf{f}_{i}-\tilde{\mathbf{y}}_{i}\right\|^{2}_{2} \\&=\min_{F} \left\|(F- \tilde{Y})^{T} U (F- \tilde{Y})\right\|_{2}^{2}  \end{aligned}  $$

where $\mathbf {f}_{i} \in \mathbb {R}^{K}$ is the to be predicted probability likelihood on the *i*-th protein, *F*=[**f**_*i*_,**f**_2_,…,**f**_*n*_] is the predictions on *n* proteins, $\tilde {Y}=\left [\tilde {\mathbf {y}}_{1},\tilde {\mathbf {y}}_{2},\ldots,\tilde {\mathbf {y}}_{n}\right ]$ is the likelihood matrix for confirmed labels along with the estimated missing labels on *n* proteins, *U* is an *n*×*n* diagonal matrix with *U*_*ii*_=1 if *i*≤*l*, and *U*_*ii*_=0 otherwise.

Proteins with similar amino acid sequences are likely to share the same functions. Schwikowski *et al.* [37] observed that two interacting proteins are more likely to share the same functions than two proteins with no interaction with each other. This observation is recognized as the ‘guilt by association’ rule. Inspired by the work [38] that states that the labels of an unlabeled instance can be linearly inferred from the labels of its neighbors, we introduce a smoothness term to utilize the interactions (or similarity) between proteins as:

(7) $$\begin{array}{@{}rcl@{}} \Psi_{2}(f)&=&\min_{f}\sum^{n}_{i=1} \left\|\mathbf{f}_{i}- \sum_{p_{j} \in \mathcal{N}(p_{i})}W_{ij}\mathbf{f}_{j} \right\|^{2}_{2}  \\ &=&\min_{F} \left\| F^{T}(I-W)^{T}(I-W) F \right\|_{2}^{2} \\ &&s.t. \sum_{j=1}^{n} W_{ij}=1  \end{array} $$

where $\mathcal {N}(p_{i})$ is the set of proteins interacting with *p*_*i*_, *W*_*ij*_ is the weight of the interaction (similarity) between proteins *i* and *j*, and *I* is an *n*×*n* identity matrix. Our motivation to minimize Eq. (7) is three-fold: (i) if two proteins *i* and *j* are quite similar to one another (or *W*_*ij*_ is large), then the margin between **f**_*i*_ and **f**_*j*_ should be small, otherwise there is a big loss; (ii) if protein *i* has missing labels and its interacting partners do have those labels, then we can leverage this information to assist the replenishing process of the missing labels for protein *i*; (iii) if protein *i* is completely unlabeled, its labels can be predicted using the labels of its partners. Alternative ways (i.e., based on functional connectivity or homology between proteins) to transfer labels among proteins have been suggested in the literature (see [5,39-41]). These methods can also be adapted to replace Eq. (7). Since our work focuses on how to replenish the missing labels and how to predict protein functions using incomplete hierarchical labels, how to more efficiently utilize the guilt-by-association rule and how to reduce noise in PPI networks to boost the accuracy (i.e., by enhancing the functional content [42], or by incorporating additional data sources [5,15,16]), is out of scope.

Based on Eq. (6) and Eq. (7), the objective function to be minimized by the PILL algorithm is:

(8) $$ {\fontsize{8}{6}\begin{aligned} \Psi(F)=&\,tr\left(\left(F- \tilde{Y}\right)^{T} U \left(F- \tilde{Y}\right)\right)\\ &+ \;\lambda tr\left(F^{T}(I-W)^{T} (I-W) F\right) \end{aligned}}  $$

where *λ*>0 is a scaler parameter that balances the importance of the empirical loss and the smoothness loss.

## Results and discussion

### Datasets and experimental setup

We report the results on three PPI networks, namely CollingsPPI, KroganPPI, and ScPPI. We annotated proteins in these networks according to MIPS FunCat [32] and Gene Ontology [33] (Biological Process non-IEA terms) respectively. The statistic of these preprocessed datasets is listed in Table 1. The CollingsPPI dataset, for example, has 1620 proteins labeled with 168 distinct GO labels and 176 FunCat labels; these proteins in total are labeled with 22,023 GO labels and 13,320 FunCat labels, and on average each protein has about 13.59 GO labels and 8.22 FunCat labels. More details on these datasets and experimental setup are provided in the Additional file 1. The label vector of proteins implicitly encodes the hierarchical relationship among labels. For example, suppose the entry index corresponding to ‘01.01’ in label vector $\mathbf {y}_{i} \in \mathbb {R}^{K}$ is *t*, and the entry index corresponding to ‘01’ (the ancestor function label of ‘01.01’) is *s*, if *y*_*it*_=1, then *y*_*is*_=1.

**Table 1:** **Dataset statistics**

**Dataset**	***#*** **Proteins**	***#*** **FunCat labels**	***#*** **GO labels**	**Avg** ***±*** **Std(FunCat)**	**Avg** ***±*** **Std(GO)**
CollinsPPI	1620	176 (13320)	168 (22023)	8.22 ±5.60	13.59 ±8.28
KroganPPI	2670	228 (20384)	241 (32639)	7.63 ±5.81	12.22 ±8.83
ScPPI	5700	305 (36909)	372 (61048)	6.48 ±5.71	10.71 ±8.83

‘ *#*Proteins’ represents the number of proteins in a dataset, ‘ *#*FunCat Labels’ describes the number of distinct FunCat labels of these proteins and the number in the bracket represents the total number of FunCat labels on all these proteins, ‘ *#*GO Labels’ represents the number of distinct GO labels of these proteins and the number in the bracket represents the total number of GO labels on all these proteins, ‘Avg ±Std(FunCat)’ represents the average number of FunCat labels for a protein in a dataset and the standard deviation, ‘Avg ±Std(GO)’ represents the average number of GO labels for a protein in a dataset and the standard deviation.

There are no off-the-shelf proteomic datasets that can be directly used to test the performance of the solution of the incomplete labels problem, although this problem is practical and common in real world scenarios. To address this issue, we assume the labels of the currently labeled proteins are complete and randomly mask some of the ground truth leaf functions of a protein; these masked functions are considered as missing for this protein.

For representation, we use *m* as the number of missing functions of a protein. For example, if a protein has 10 functional labels, *m*=3 means that three functional labels are masked for this protein. If a protein does not have more than *m* labels, we do not mask all the available labels and ensure it has one function label. A small number of proteins in these networks doesn’t have any label; we keep these proteins in the network to retain the network’s structure, but do not test on them. We introduce *N*_*m*_ to represent how many labels are missing for all the proteins for a given setting of *m*.

### Comparing methods and evaluation metrics

We compare PILL against ProDM [14], ProWL [29], LkNN [22], TPR [7], MLR-GL [30], CIA [26], and Naive [9]. ProDM and ProWL are designed to replenish the missing labels and to predict protein functions using partially labeled proteins; neither explicitly considers the hierarchical relationship among function labels. LkNN utilizes *LinSim* in Eq. (1) to predict the functions of unlabeled proteins. TPR uses the true path rule (or hierarchical relationship) in label hierarchies to refine the predictions of binary classifiers trained for each label. We use the weighted version, TPRw, for the experiments. MLR-GL uses partially labeled instances in the training set to predict the labels of unlabeled instances. CIA is an iterative algorithm that uses function correlations based on Cosine similarity to infer protein functions. Naive, which ranks functional labels based on their frequencies, is a baseline approach in the community-based critical assessment of protein function annotation [9]. It is reported that very few methods performed above the Naive method. Therefore, we take the Naive method as a comparing method for reference. More details about the implementations and parameter settings of these methods are reported in the Additional file 1.

The performance of protein function prediction can be evaluated according to different criteria, and the choice of evaluation metrics differentially affects different prediction algorithms [9,29]. For a fair and comprehensive comparison, we used five representative metrics, namely *MacroF1*, *MicroF1*, *AvgROC*, *RankingLoss* and *Fmax*. These evaluation metrics are extensively applied to evaluate the performance of multi-label learning algorithms and protein function prediction [9,21,29]. The formal definition of these metrics is provided in the Additional file 1. To keep consistency across all evaluation metrics, we use *1-RankLoss* instead of *RankingLoss*. Thus, the higher the value, the better the performance is for all the used metrics. These metrics evaluate the performance of function prediction in different aspects, and thus it is difficult for an algorithm to outperform another technique on all the metrics.

### Replenishing missing function labels

In this section, we conduct experiments to study the performance of PILL on replenishing missing annotations of *n* hierarchically and partially labeled proteins. In the experiments, we consider all the proteins in the dataset as training and testing data. The experimental results with *m*=1,3,5 on CollingsPPI with respect to the FunCat labels are reported in Table 2 (the best and comparable results are in **bold** font, with statistical significance examined by a pairwise *t*-test at 95% significance level). Other results on CollingsPPI, KroganPPI and ScPPI are reported in Tables S1-5 of the Additional file 1. For each setting of *m*, the experiments are repeated 20 times. In each run, the masked labels of a protein are randomly chosen from the leaf function labels of the same protein, and these masked labels are considered as missing for testing. If *s* is a non-leaf function label of a protein, whenever its descendant function labels are all missing (or masked), *s* turns to be a leaf function label and can be masked for this protein.

**Table 2:** **Results of replenishing missing labels on CollinsPPI wrt**

**Metric**	***m(N***_***m***_ ***)***	**PILL**	**ProDM**	**ProWL**	**LkNN**	**TPRw**	**Naive**
MicroF1	1(1526)	**93.91 ±0.11**	83.30 ±0.30	90.31 ±0.08	44.07 ±0.14	50.00 ±0.12	29.00 ±0.01
	3(4330)	**81.70 ±0.29**	72.16 ±0.77	78.38 ±0.23	41.61 ±0.16	43.60 ±0.18	29.77 ±0.13
	5(6580)	**70.53 ±0.31**	60.10 ±1.01	66.61 ±0.16	37.54 ±0.21	36.79 ±0.13	30.09 ±0.06
MacroF1	1(1526)	**89.29 ±0.25**	69.53 ±0.40	85.75 ±0.34	34.23 ±0.21	43.33 ±0.15	4.70 ±0.01
	3(4330)	**70.19 ±0.63**	60.78 ±1.73	69.03 ±0.46	29.23 ±0.48	35.45 ±0.39	5.06 ±0.04
	5(6580)	**55.32 ±0.94**	45.37 ±1.90	52.95 ±0.59	24.12 ±0.62	27.06 ±0.75	5.13 ±0.05
AvgROC	1(1526)	**99.47 ±0.01**	97.44 ±0.06	98.27 ±0.09	66.14 ±0.05	69.67 ±0.18	49.44 ±0.00
	3(4330)	**97.77 ±0.16**	93.86 ±0.44	93.35 ±0.18	64.86 ±0.11	64.93 ±0.21	49.44 ±0.00
	5(6580)	**94.64 ±0.33**	87.03 ±1.04	86.24 ±0.49	63.25 ±0.34	60.41 ±0.36	49.44 ±0.00
1-RankLoss	1(1526)	**99.43 ±0.03**	96.80 ±0.04	98.55 ±0.05	69.38 ±0.04	55.75 ±0.14	79.33 ±0.04
	3(4330)	**97.58 ±0.11**	92.15 ±0.26	94.62 ±0.17	66.09 ±0.27	46.90 ±0.47	76.72 ±0.22
	5(6580)	**94.55 ±0.27**	86.63 ±0.67	89.30 ±0.25	59.65 ±0.65	36.88 ±0.41	74.52 ±0.41
Fmax	1(1526)	**90.88 ±0.07**	76.28 ±0.31	80.49 ±0.34	42.74 ±0.09	58.43 ±0.36	28.32 ±0.00
	3(4330)	**76.82 ±0.10**	67.39 ±0.84	66.14 ±0.29	42.16 ±0.25	51.12 ±0.50	27.93 ±0.01
	5(6580)	**66.11 ±0.50**	56.26 ±4.01	57.76 ±0.52	40.39 ±0.37	44.01 ±0.53	27.04 ±0.00

FunCat labels. *m* is the number of missing labels for a protein and *N*_*m*_ in the bracket is the total number of missing labels for all the proteins. The numbers in **boldface** denote the best performance.

From the results reported in these Tables, we can observe that PILL outperforms other competitive methods across all the evaluation metrics in most cases. In summary, out of 90 configurations (3 datasets × 2 kinds of labels × 5 evaluation metrics × 3 settings of *m*), PILL outperforms ProDM 85.56% of the cases, outperforms ProWL 91.11% of the cases, ties with them 4.44% and 4.44% of the cases, and loses to them in 5.56% and 4.44% of the cases, respectively. PILL outperforms LkNN, TPRw and Naive in all configurations. Taking *MacroF1* on CollingsPPI annotated with FunCat labels, for example, PILL on average is 23.30% better than ProDM, 4.27% better than ProWL, 147.33% better than LkNN, and 106.61% better than TPRw. These results corroborate the effectiveness of PILL on replenishing the missing labels.

PILL largely outperforms ProDM and ProWL, even if the latter two also leverage correlation between function labels and the interaction between proteins. The reason is that ProDM and ProWL use the Cosine based similarity to define the correlation between function labels, and they do not explicitly make use of the hierarchical relationship among labels. Since the label vector implicitly encodes the hierarchical relationship of labels to some extent, ProDM and ProWL can achieve a result comparable (or a slightly better) to PILL in few cases.

LkNN and TPRw explicitly utilize the hierarchical relationship among labels, but they are not able to compete with PILL, ProDM and ProWL. The cause is two fold: (i) LkNN and TPRw assume that the labels of the labeled proteins are complete, and they use partially labeled proteins to predict missing labels without estimating the missing labels in advance; (ii) they do not utilize the flat relationships among function labels. Naive ranks the functional labels according to their frequency and sets the frequency as the predicted probability for the labels. Since the missing labels are ‘leaf’ functional labels, and their frequencies are smaller than the ‘non-leaf’ functional labels, Naive achieves the lowest *AvgROC* and *MacroF1* scores, a medium *1-RankLoss* score, and almost the lowest *Fmax* and *MicroF1* scores among the comparing methods. Naive performs better than some methods in few cases, but it is outperformed by PILL by a large margin across all the evaluation metrics. These results show that PILL can exploit the hierarchical and flat relationships among labels to boost the performance of protein function prediction.

*Real Life Examples*: Another experiment is performed to study the ability of PILL on providing hypothetical missing labels. In particular, we use the GO terms associations (download date: 2014-02-01) of *S. Cerevisiae* to annotate the proteins in ScPPI (here we do not apply the filter process to remove the too specific and too general labels as in the previous experiments, and these 5700 proteins were annotated with 2381 distinct biological process labels). We use PILL to replenish the missing labels of these proteins. There are 117 proteins in ScPPI having new labels in the updated GO terms annotations [33](download date: 2014-06-01), and there are 451 newly appended labels for these proteins. We choose the top 50 function labels (from 2381 distinct labels) as the hypothetical labels for each of these proteins. We observe PILL can correctly replenish 30.38%(137/451) missing labels, and if we append the ancestor labels of these 137 labels to these 117 proteins, 40.80%(184/451) labels are correctly replenished. These proteins are provided in Additional file 2, and some examples are reported in Table 3. In the table, the original (date before 2014-02-01) GO labels and the replenished ones of a protein, the support reference’s PMID, the GO term annotation (or protein label association) added date and the GO term annotation evidence code are all listed. Evidence code indicates the type of evidence that supports the go term annotation, ‘IMP’ is Inferred from Mutant Phenotype, ‘IDA’ is Inferred from Direct Assay, ‘IGI’ is Inferred from Genetic Interaction, and ‘ISS’ is Inferred from Sequence or Structural Similarity. These real life examples demonstrate PILL can confidently provide hypothetical missing labels from a large number of candidate labels.

**Table 3:** **Examples of replenished labels for proteins by PILL and their support references**

**Protein**	**Original label**	**Replenished label**	**Evidence code**	**PMID**	**Date**
YOR206W	GO:0042255, GO:0000054	GO:0042273	IMP	PMID:23209026	2014-05-02
YGR104C	GO:0045944,GO:0051123,GO:0001113	GO:0006353, GO:0006369	IMP	PMID:23476016	2014-03-28
YML074C	GO:0051598,GO:0018208,GO:0000412	GO:0006334	IDA	PMID:24297734	2014-05-23
YBL102W	GO:0006895	GO:0042147	IGI	PMID:10406798	2014-03-14
YJL102W	GO:0006414	GO:0032543	ISS	PMID:19716793	2014-04-02

‘Original label’ is the available labels of a protein before 2014-02-01, and ‘Replenished label’ is the replenished label by PILL, ‘Evidence code’ is the type of evidence that supports the go term annotation (or protein label association), ‘Reference’ is the PMID of the support reference for this go term annotation, and ‘Date’ is the date this go term annotation was added.

### Predicting functions for unlabeled proteins

We performed another set of experiments to test the performance of PILL on predicting functions for completely unlabeled proteins using partially labeled proteins. In these experiments, *l*<*n* proteins are partially labeled, and the remaining *n*−*l* proteins are completely unlabeled. PILL cannot estimate the likelihood of missing labels for these proteins, since no labels are available. PILL makes use of *C*_*m*_ and the PPI information to replenish the missing labels for the partially labeled proteins, and then the initially available labels together with the replenished ones can be transferred to these completely unlabeled proteins. We randomly select 70% of the proteins as the training set and the remaining ones as testing set. For each protein in the training set, we simulate the setting (*m*=3) of incomplete labels as in the previous experiments. The experimental results with respect to CollingsPPI are reported in Table 4 (other results on CollingsPPI, KroganPPI and ScPPI are reported in Tables S6-10 of the Additional file 1). All the results in these tables are the average of 20 independent runs; in each run, the training and testing sets are randomly partitioned, and the masked leaf function labels in the training set are randomly selected as in the previous experiments.

**Table 4:** **Prediction results on complete unlabeled proteins of CollinsPPI wrt**

**Metric**	**PILL**	**ProDM**	**ProWL**	**LkNN**	**TPRw**	**MLR-GL**	**CIA**	**Naive**
MicroF1	**47.05 ±1.24**	34.44 ±2.11	37.58 ±1.24	32.06 ±1.21	33.79 ±1.62	28.53 ±0.87	33.59 ±2.19	25.47 ±0.46
MacroF1	**29.29 ±3.02**	16.60 ±4.67	26.11 ±0.90	20.30 ±1.51	22.74 ±1.96	20.58 ±1.14	23.43 ±1.94	1.97 ±0.04
AvgROC	**77.48 ±2.25**	64.37 ±1.27	56.97 ±1.08	64.45 ±1.82	61.54 ±1.67	64.29 ±1.08	57.18 ±1.24	49.74 ±1.26
1-RankLoss	**82.64 ±0.41**	77.90 ±3.66	64.57 ±1.82	50.10 ±2.37	42.49 ±2.02	39.36 ±1.08	64.07 ±3.23	76.60 ±0.67
Fmax	**56.57 ±1.12**	26.05 ±7.42	16.22 ±1.00	41.60 ±0.67	47.42 ±2.45	40.32 ±0.74	32.19 ±1.96	27.52 ±0.35

FunCat labels.

From these tables, we can observe that PILL achieves the best results among all the comparing methods. PILL, ProDM and ProWL take into consideration the incomplete annotation in the training set, and they often outperform LkNN, TPRw, and CIA. MLR-GL considers the incomplete annotation in the training set, but it does not explicitly use the hierarchical relationship between labels. Thus, it loses to the competing algorithms. TPRw post-processes the predictions of binary classifiers according to the true path rule, and sometimes it achieves comparable results to PILL. For a fair comparison with the other algorithms, we do not apply the true path rule to refine the predictions made by PILL in Eq. (8). Naive, a baseline and yet competitive approach in community based critical assessment of function annotation [9], performs above the comparing methods with respect to some metrics (i.e., *1-RankLoss* and *Fmax*, which are more favorable to the frequency based ranking than other metrics). However, Naive is outperformed by PILL by a large margin. Given the superior performance of PILL to Naive, PILL can serve as a valuable method for protein function annotation.

From these results, we can draw the conclusion that it is important to utilize the relationships (including hierarchical and flat ones) among labels, and to explicitly consider the incomplete label problem in protein function prediction. These results also corroborate the effectiveness of PILL on predicting protein functions on unlabeled proteins using hierarchical incomplete labeled proteins.

### The benefit of using hierarchical and flat relationships between labels

**Figure 3:**
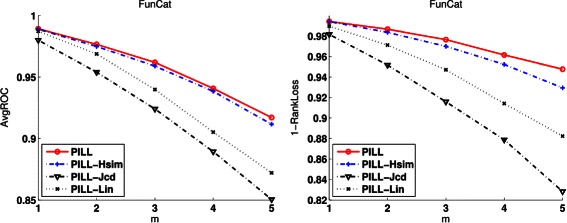
**The benefit of using function correlation defined by** ***ComSim*** **,** ***HSim*** **,** ***LinSim*** **and Jaccard coefficients on the proteins in** ***CollingsPPI*** **annotated with FunCat labels.** PILL uses *ComSim*, PILL-HSim utilizes *Hsim*, PILL-Lin uses *LinSim* and PILL-Jcd is based on Jaccard coefficients.

We did another kind of experiments to investigate the benefit of using the proposed *ComSim* in Eq. (4). *ComSim* not only takes into account the hierarchical relationship, but also the flat relationship between function labels. For comparison, we introduce three variants of PILL: (i) PILL-Jcd is PILL with the function correlation defined by the Jaccard coefficient; (ii) PILL-Hsim is PILL with the function correlation defined by *HSim* using the *shared* ancestors in Eq. (3). (iii) PILL-Lin is PILL with the function correlation defined by *LinSim* using the *common* ancestors in Eq. (2); From these variants, it is easy to find that PILL-Jcd does not explicitly use the hierarchical relationship between labels, and PILL-Hsim and PILL-Lin do not use the flat relationship between labels. We use the task of replenishing missing labels to study the difference among PILL, PILL-Hsim, PILL-Lin, and PILL-Jcd. The experimental results (*AvgROC* and *1-RankLoss*) on CollingsPPI annotated with FunCat labels are reported in Figure 3. The results on CollingsPPI and KroganPPI with respect to other evaluation metrics are reported in Figures S3-5 of the Additional file 1.

From these figures, we can observe that using *LinSim*, *HSim* (a variant of *LinSim*) or the Jaccard coefficient separately often cannot achieve results comparable to PILL. PILL-Hsim based on *HSim* uses the *shared* ancestor labels, and performs better than PILL-Lin based on *LinSim*, which utilizes the *common* ancestor labels. This fact supports our motivation to define the *HSim* using the *shared* ancestor labels instead of the *common* ones. The superiority of PILL over PILL-Jcd indicates that hierarchical relationships between function labels are more important than flat relationships. The larger the number of missing labels, the larger the performance margin between PILL and PILL-Jcd is. These observations support our motivation to use *ComSim* to exploit both the hierarchical and flat relationships between labels to boost the performance.

### The benefit of using function correlation and guilt by association rule

**Figure 4:**
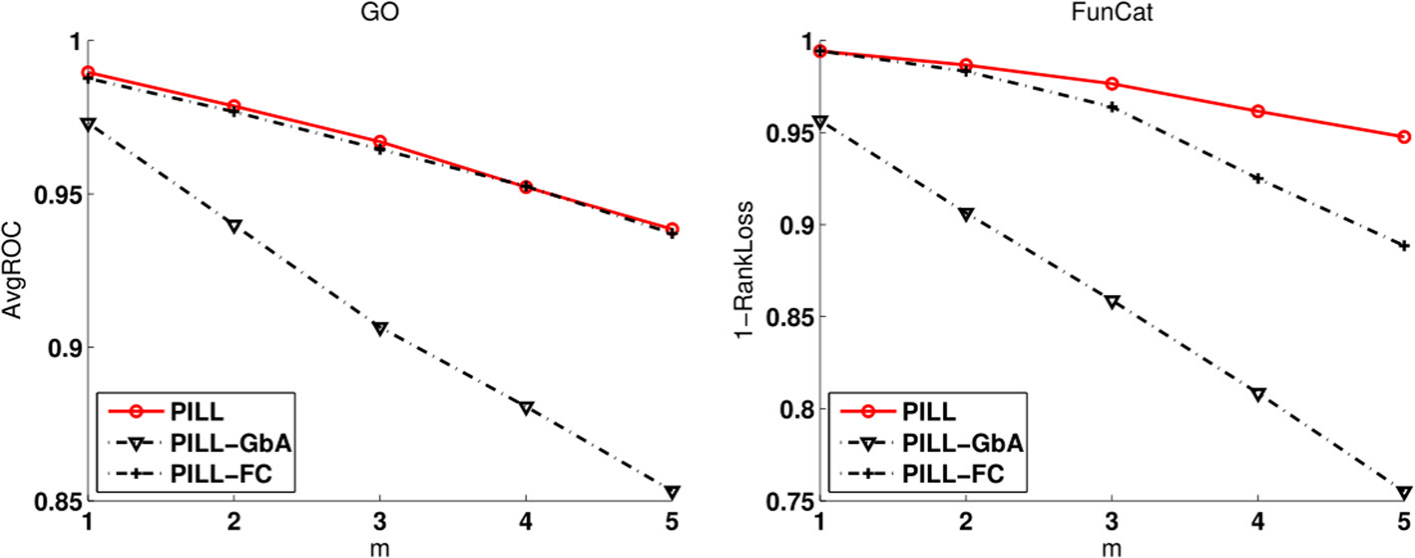
**The benefit of using function correlation and Guilt by Association rule on the proteins in** ***CollingsPPI*** **annotated with FunCat labels.** PILL-FC only uses the function correlation between function labels, PILL-GbA only uses the guilty by association rule, and PILL uses both the function correlation and the guilt by association rule.

We conducted experiments to study the benefit of using function correlations and the guilt by association rule. We define two variants of PILL: (i) PILL-FC just utilizes the estimated $\tilde {Y}$, without using the second term (‘Guilt by Association’ rule) in Eq. (8), and (ii) PILL-GbA just uses the second term in Eq. (8) and does not use function correlations to estimate the missing labels. The recorded results (*AvgROC* and *1-RankLoss*) on CollingsPPI with respect to FunCat labels are given in Figure 4. The results on CollingsPPI and KroganPPI with respect to other evaluation metrics are reported in Figure S6-8 of the Additional file 1.

From these results, we can say that using the function correlation or the guilt by association rule separately cannot replenish the missing labels as well as PILL. PILL-FC often achieves better results than PILL-GbA. This fact shows that using function correlation alone can replenish the missing labels to some extent. From these results, we can draw the conclusion that both the function correlations and the guilt by association rule are beneficial to replenish the missing labels of proteins, and PILL can jointly utilize these two components to boost the performance of protein function prediction.

## Conclusions and future work

In this article, we investigated the seldom studied (but yet important and practical) problem of protein function prediction with partial and hierarchical labels. We proposed an approach, PILL, to replenish the missing labels of partially labeled proteins and to predict functions for completely unlabeled proteins. Our empirical study shows that PILL outperforms a range of related methods and PILL can confidently provide hypothetical missing labels from a large number of candidate labels.

Some methods have been proposed to explore node-based (or edge-based) similarities to measure the semantic similarity of functional labels [4,43]. These methods capture different characteristics of the ontology structure and correlate with protein sequence similarity, PPI networks, and other types of genomic data to some extent. As part of our future work, we are interested in integrating these characteristics of the functional label structure to accurately estimate the missing labels and predict functions for unlabeled proteins.

## Additional files

Additional file 1
**Supplementary file of ‘Predicting protein function using incomplete hierarchical labels’.** This pdf file includes the example of incomplete hierarchical label problem in the GO hierarchy, the function label relationship statistics and similarity comparison on GO, the definition of evaluation metrics, parameters setting and additional experimental results. This file can be accessed by the link in the reference [44].

Additional file 2
**Real life examples.** This txt file includes the real life examples of correctly replenished labels for proteins by PILL. This file can be accessed by the link in the reference [44].
