# Supplementary material for: Predicting protein functions using incomplete hierarchical labels
Source: BMC Bioinformatics. 2015 Jan 16;16:1. doi: 10.1186/s12859-014-0430-y (PMC4384381; doi:10.1186/s12859-014-0430-y)
Supplement: Additional file 1 — Supplementary file of ‘Predicting protein function using incomplete hierarchical labels’. This pdf file includes the example of incomplete hierarchical label problem in the GO hierarchy, the function label relationship statistics and similarity comparison on GO, the definition of evaluation metrics, parameters setting and additional experimental results. This file can be accessed by the link in the reference [44]. [file 12859_2014_430_MOESM1_ESM.pdf]

Supplementary file of 'Predicting protein functions using incomplete hierarchical labels'

Guoxian Yu[1,2,*], Hailong Zhu[1,*] and Carlotta Domeniconi[3]
[1]Department of Computer Science, Hong Kong Baptist University, Hong Kong
[2]College of Computer and Information Sciences, Southwest University, Chongqing, China
[3]Department of Computer Science, George Mason University, VA, USA
[*]Contact: guoxian85@gmail.com, hlzhu@comp.hkbu.edu.hk

January 27, 2015

Incomplete hierarchical label problem in the GO hierarchy

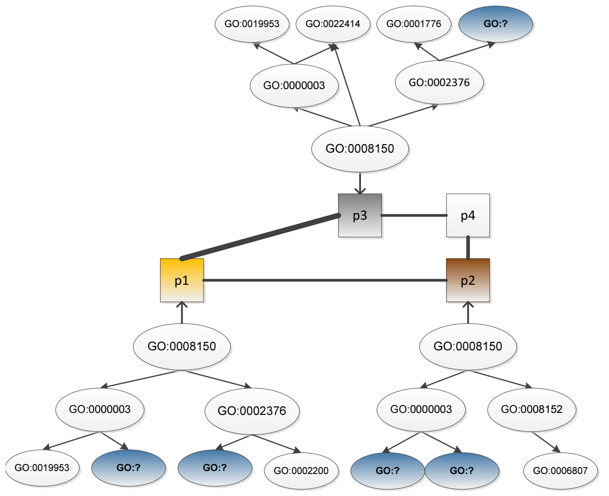

Figure S1 Illustration of incomplete hierarchical labels for proteins labeled with Gene Ontology terms. A rectangle represents a protein (pi, $i \in \{1, 2, 3\}$); an ellipse denotes a function label, and a undirected line between rectangles captures a protein-protein interaction (the more reliable the interaction is, the thicker the line is). All the functional labels (including the missing function labels denoted by colored ellipses labeled with question marks '?') in the ellipses should be associated with the proteins, but only the functional labels in the white ellipses are known. For better visualization, other functional labels (i.e., 'GO:0001906' and 'GO:0009987', which are not ground-truth labels for these proteins), are not plotted in the Figure.

In the main paper, we illustrated the scenario of incomplete hierarchical labels of proteins under MIPS FunCat labels. The corresponding scenario under GO labels is shown in Fig. S1. The missing labels are leaf function labels. If a non-leaf function label of a protein is missing, we can directly append this function label to the protein from its descendant function labels. Each hierarchy of non-leaf and leaf function labels is defined with respect to a single protein. For example, 'GO:0000003' is a non-leaf label of $p1$; it has a descendant function label 'GO:0019953'. If 'GO:0000003' is missing for $p1$, but 'GO:0019953' is not missing for $p1$, we can directly append 'GO:0000003' to the label set of $p1$. In contrast, 'GO:0000003' is a leaf function label of $p2$, since none of its descendant functions is known to be annotated with $p2$.

Function label relationship statistics and similarity comparison

In the main paper, we reported the statistics of label relationships and four label correlations (defined as Cosine similarity, Jaccard coefficient, Lin's similarity, and our proposed *ComSim*) on the proteins of *S. Cerevisiae* labeled with FunCat labels. Fig. S2 gives the distributions of the relationships parent-child, grandparent-grandson, and uncle-nephew for the proteins in *S. Cerevisiae* (labeled with GO Biological Process terms).

GO organizes the function labels in a directed acyclic graph; unlike FunCat, there is no strict family tree relationships in GO. For example, 'GO:0008150' is a parent function label of 'GO:0000003' and 'GO:0022414', and 'GO:0000003' is also a parent function label of 'GO:0022414'. For the statistics of the likelihoods of uncle-nephew, for a function label s, its parent function label set is $par(s)$, and its grandparent function label set is $gpar(s)$. We consider the children function label set (excluding $par(s)$) of $gpar(s)$ as the uncle label set $uncle(s)$ of s. We study the parent-child relationship by counting the cases in which a protein is labeled with both a function label in $par(s)$ and with s. We proceed similarly to compute the cases in which a protein is labeled with both a label in $gpar(s)$ (or $uncle(s)$) and with s. In addition, we also computes

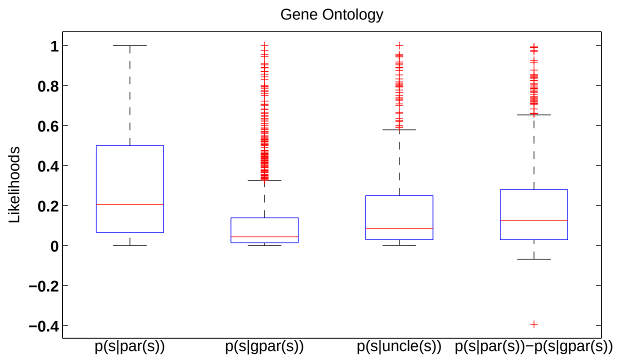

(a) Likelihoods

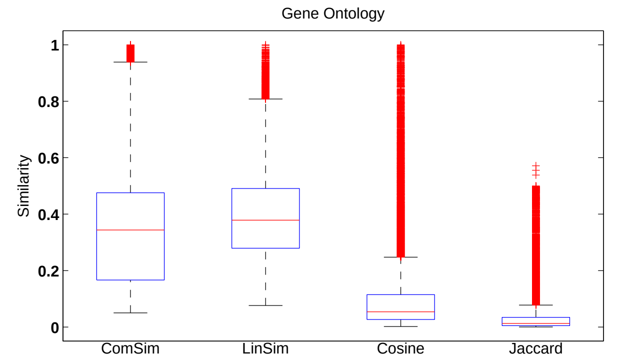

(b) Similarities

Figure S2 Label relationship statistics and four label similarities on proteins of *S. Cerevisiae* annotated with Gene Ontology Biological Process labels.

difference between $p(s|par(s))$ and $p(s|gpar(s))$ in the fourth boxplot of Fig. 2(a). A function label in GO may have several parent (or grandparent) function labels. To reveal the distribution of $p(s|par(s)) - p(s|gpar(s))$ for each s, we *average* the likelihood of $p(s|par(s))$ with respect to all its parent function labels. Similarly, we *average* the likelihood of $p(s|gpar(s))$ with respect to all its grandparent function labels. Therefore, a small portion of $p(s|par(s)) - p(s|gpar(s))$ may be negative in the fourth boxplot of Fig. 2(a). For example, 'GO:0006270' has two direct parent labels 'GO:0006261' and 'GO:0006259', and 'GO:0006261' has a parent label 'GO:0006260'. On the other hand, 'GO:0006260' is the child of 'GO:0006259'. Our statistics show 'GO: 0006270' is associated with 32 proteins, its parent 'GO:0006261' and 'GO:0006259' are associated with 99 and 429 proteins, and its grandparent 'GO:0006260' is associated with 120 proteins. In this case, $p(s|par(s)) - p(s|gpar(s))$ is smaller than 0. We observe that, if s has one parent label and one grandparent label, $p(s|par(s)) \geq p(s|gpar(s))$ still holds. The distributions obtained on the *S. Cerevisiae* proteins labeled with GO labels are similar to the distributions (FunCat labels) in the main paper.

Datasets

CollingsPPI and KroganPPI are obtained from the study [1] and the study [2], respectively. The interactions between proteins in these two networks are reliable and weighted with real values. ScPPI is a unweighted network (the weights of all physical interactions are set to 1) of *S.cerevisiae* extracted from BioGrid[1] (version 3.2.109, downloaded and processed date: 2014-02-15); it's the largest connected component of *S.cerevisiae*. As suggested and done in [3–5], the functional label which has too few member proteins is not likely to be testable in the wet lab, and thus of no interest to biologists. For proteins labeled with FunCat, we retained the function label which has at least 10 member proteins; for proteins annotated with GO, we kept the function labels that have at least 30 member proteins. We also removed the function label corresponding to 'GO:0008150' (root node of Biological Process), and '99' ('UNCLASSIFIED PROTEINS' in MIPS FunCat).

Comparing methods and evaluation metrics

We set the parameters of the comparing methods (i.e., ProDM, ProWL and MLR-GL) as the authors reported in their papers, or as provided in their code. As to LkNN, for a fair comparison, we use the same threshold (0.05) used for *LinSim* and *ComSim*. Any correlation between function labels below 0.05 is set to 0. In practice, we observe that almost all nonzero entries, when using *LinSim* or *ComSim*, are larger than 0.05; therefore, this preprocessing does not deteriorate the performance. TPRw depends on the predicted likelihoods given by the binary classifier trained for each label; to keep consistency across the other comparing methods, we use the predictions given by a neighborhood counting algorithm ([6]) as the initial likelihoods. We set both the threshold and the weight factor values of TPRw to 0.5, as suggested by the authors. In practice, CIA also used the neighborhood counting algorithm to get the initial predictions and to kick off the iteration. For PILL, we optimized $\lambda \in \{10^{-4}, 10^{-3}, \ldots, 10^{4}\}$ via 5-fold cross validation on the training set, and set $\lambda = 1$, since we observed PILL had relative stable performance when λ's values are close to 1.

[1] http://thebiogrid.org/download.php

Here, we introduce the formal definition of four representative evaluation metrics (*MacroF1*, *MicroF1*, *AvgRoc*, and *RankingLoss*) in multi-label learning [7, 8]. Some of these metrics are also used to evaluate the performance of protein function prediction [9–11].

MacroF1 is the average $F1$ scores of different labels:

$$MacroF1 = \frac{1}{K}\sum_{k=1}^{K}\frac{2p_k r_k}{p_k + r_k}$$

where K is the number of labels, p_k and r_k are the precision and recall of the k-th label, defined as:

$$p_k = \frac{TP_k}{TP_k + FP_k} \quad r_k = \frac{TP_k}{TP_k + FN_k}$$

TP_k, FP_k, and FN_k are the true positive, false positive, and false negative with respect to the k-th function label. From the definition, it can be observed *MacroF1* first calculates the F1 measures for each label, and then averages over all the labels. *MacroF1* is more affected by the performance on the labels containing fewer member proteins.

MicroF1 calculates the $F1$ measure on the predictions of different labels as a whole:

$$MicroF1 = \frac{\sum_{k=1}^{K} 2p_k r_k}{\sum_{k=1}^{K} p_k + r_k}$$

MicroF1 is more affected by the performance on labels containing more member proteins.

Average ROC (AvgROC) score is a function centric evaluation metric, it averages the receiver operation curve (ROC) score of each function. The ROC score is calculated as the area under the ROC curve, which plots the true positive rate (sensitivity) as a function of the false positive rate (1-specificity) under different classification thresholds. It measures the overall quality of the ranking induced by the classifier, instead of the quality of a single value of the threshold in that ranking.

Ranking loss evaluates the average fraction of label pairs that are not correctly ranked:

$$RankLoss = \frac{1}{u}\sum_{i=l+1}^{N}\frac{1}{|\mathcal{Y}_i||\bar{\mathcal{Y}}_i|}|\{(k_1, k_2) \in \mathcal{Y}_i \times \bar{\mathcal{Y}}_i | F(i, k_1) \leq F(i, k_2)\}|$$

where $\mathcal{Y}_i$ is the function set of protein i, and $\bar{\mathcal{Y}}_i$ is the complement set of $\mathcal{Y}_i$, $F(i, k_1)$ is the predicted likelihood for the i-th protein having the k_1-th function. The smaller the value of *RankLoss*, the better the performance is.

Fmax is a protein centric evaluation metric used in the community-based critical assessment of protein function annotation (CAFA) [12], *Fmax* is an F-measure computed as:

$$Fmax = \max_t \frac{2p(t)r(t)}{p(t) + r(t)}$$

where $p(t) = \frac{1}{m(t)}\sum_{i=1}^{m(t)} p_i(t)$ is the the precision at threshold $t \in [0, 1]$, $p_i(t)$ is the precision on the i-th protein, $m(t)$ is the number of proteins on which at least one prediction was made above the threshold t, $r(t) = \frac{1}{u}\sum_{i=1}^{u} r_i(t)$ is the recall across u proteins at threshold t.

To keep consistency with other evaluation metrics, we report *1-RankLoss* instead of *RankLoss*. Thus, the higher the value of these evaluation metrics, the better the performance is. *MacroF1* and *MicroF1* require the predicted likelihood score vector $\mathbf{f}_i$ to be a binary indicator vector. We consider the functions corresponding to the r largest values of $\mathbf{f}_i$ as the functions of the i-th protein. For the experiment on replenishing the missing labels, r is determined by the number of ground-truth functions of a protein. For the experiments on predicting the functions of unlabeled proteins, r is set to the average number of functions (round to the next integer) of all proteins. From Table 1 in the main paper, for CollingsPPI, r is set to 14 (GO labels) and to 9 (FunCat labels); for KroganPPI r is set to 13 (GO labels) and 8 (FunCat labels); and for ScPPI r is set to 11 (GO labels) and 7 (FunCat labels).

Replenishing missing labels

In the main paper, we reported only the results on replenishing missing labels on CollingsPPI labeled with GO labels. Here, in Tables S1-S5, we give the results ($m = 1, 3, 5$) on replenishing missing labels on CollingsPPI, KroganPPI and ScPPI labeled with GO and FunCat labels, respectively.

Predicting functions for unlabeled proteins

We provide the results of predicting functions for completely unlabeled proteins using partially labeled proteins in the training set of CollingsPPI (GO labels), KroganPPI (FunCat and GO labels), and ScPPI(FunCat and GO labels) in Tables S6-S10.

The benefit of using hierarchical and flat relationships between labels

In the main paper, we reported the results (*AvgROC* and *1-RankLoss*) of PILL, PILL-Hsim, PILL-Lin, and

Table S1 Results of replenishing missing labels on *CollinsPPI* wrt. GO labels. m is the number of missing labels for a protein and N_m in the bracket is the total number of missing labels for all the proteins. The numbers in **boldface** denote the best performance.

Metric	$m(N_m)$	PILL	ProDM	ProWL	LkNN	TPRw	Naive
MicroF1	1(1440)	**96.30±0.09**	90.25±0.09	91.21±0.08	56.92±0.07	57.88±0.04	48.86±0.20
	3(3568)	**88.63±0.16**	82.32±0.13	84.85±0.09	55.42±0.08	55.23±0.06	47.30±0.21
	5(4696)	**82.04±0.07**	76.05±0.06	77.53±0.10	54.47±0.06	52.31±0.09	44.47±0.00
MacroF1	1(1440)	**92.63±0.27**	85.75±0.14	86.28±0.16	46.06±0.20	50.92±0.14	9.37±0.04
	3(3568)	**78.66±0.47**	73.35±0.16	73.12±0.31	42.67±0.17	44.17±0.37	9.22±0.04
	5(4696)	**66.75±0.24**	61.93±0.17	60.13±0.35	39.68±0.39	38.29±0.15	8.92±0.00
AvgROC	1(1440)	**98.96±0.02**	96.86±0.06	97.33±0.05	74.41±0.10	75.30±0.07	49.65±0.00
	3(3568)	**96.70±0.21**	93.92±0.26	94.46±0.13	72.71±0.16	71.86±0.14	49.65±0.00
	5(4696)	**93.85±0.30**	90.56±0.43	92.16±0.32	71.40±0.25	68.76±0.09	49.65±0.00
1-RankLoss	1(1440)	**99.05±0.03**	95.63±0.03	96.71±0.04	64.46±0.05	61.83±0.04	80.21±0.02
	3(3568)	**96.70±0.12**	91.83±0.11	94.25±0.05	62.34±0.16	57.70±0.09	79.20±0.02
	5(4696)	**94.00±0.26**	88.48±0.05	91.29±0.12	60.78±0.27	53.23±0.18	77.34±0.07
Fmax	1(1440)	**93.99±0.06**	86.39±0.05	78.46±0.17	53.41±0.04	64.15±0.07	45.93±0.15
	3(3568)	**88.61±0.04**	80.92±0.07	75.16±0.12	53.11±0.09	61.32±0.11	44.36±0.62
	5(4696)	**85.89±0.04**	78.16±0.14	72.50±0.09	52.62±0.06	58.54±0.09	43.13±0.00

Table S2 Results of replenishing missing labels on **KroganPPI** wrt. GO labels. m is the number of missing labels for a protein and N_m in bracket is the total number of missing labels for all the proteins. The numbers in **boldface** denote the best performance.

Metric	$m(N_m)$	PILL	ProDM	ProWL	LkNN	TPRw	Naive
MicroF1	1(2212)	**95.26±0.05**	94.11±0.04	90.09±0.09	37.23±0.05	36.48±0.06	45.13±0.04
	3(5362)	**87.91±0.10**	85.38±0.05	84.01±0.11	36.50±0.05	35.21±0.08	42.83±0.06
	5(7047)	**82.39±0.04**	78.98±0.02	76.88±0.11	35.94±0.07	33.90±0.08	40.83±0.00
MacroF1	1(2212)	**91.90±0.06**	91.06±0.10	85.73±0.26	25.43±0.17	29.17±0.06	6.45±0.01
	3(5362)	**78.74±0.10**	75.76±0.17	73.49±0.33	24.01±0.13	25.49±0.12	6.21±0.02
	5(7047)	**67.30±0.19**	61.89±0.21	59.16±0.35	22.84±0.11	22.32±0.16	5.93±0.00
AvgROC	1(2212)	**98.94±0.03**	98.21±0.02	97.36±0.04	64.25±0.03	64.10±0.05	49.57±0.00
	3(5362)	**97.41±0.08**	96.37±0.04	95.53±0.04	63.41±0.02	62.05±0.07	49.57±0.00
	5(7047)	**95.17±0.21**	94.24±0.33	93.22±0.28	62.69±0.09	60.28±0.07	49.57±0.00
1-RankLoss	1(2212)	**98.93±0.02**	98.32±0.02	95.98±0.04	42.81±0.03	39.14±0.03	79.94±0.03
	3(5362)	**97.57±0.09**	95.69±0.06	94.48±0.07	41.88±0.03	36.74±0.04	78.84±0.04
	5(7047)	**95.74±0.18**	92.50±0.16	91.91±0.24	41.15±0.09	34.60±0.12	77.50±0.06
Fmax	1(2212)	**93.63±0.02**	92.62±0.02	76.26±0.10	36.31±0.05	41.29±0.04	41.73±0.00
	3(5362)	**88.45±0.02**	87.44±0.01	72.55±0.13	36.09±0.05	40.03±0.04	39.41±0.07
	5(7047)	**85.91±0.02**	85.00±0.05	70.41±0.19	35.84±0.11	38.68±0.08	39.36±0.00

Table S3 Results of replenishing missing labels on **KroganPPI** wrt. FunCat labels. m is the number of missing labels for a protein and N_m in bracket is the total number of missing labels for all the proteins. The numbers in **boldface** denote the best performance.

Metric	$m(N_m)$	PILL	ProDM	ProWL	LkNN	TPRw	Naive
MicroF1	1(2340)	**91.15±0.05**	90.27±0.06	88.49±0.12	26.29±0.08	29.51±0.03	29.48±0.04
	3(6612)	**76.21±0.12**	74.01±0.11	75.64±0.23	25.40±0.15	26.86±0.10	29.81±0.07
	5(10009)	**64.47±0.18**	62.36±0.15	63.78±0.15	24.00±0.22	23.87±0.15	29.63±0.15
MacroF1	1(2340)	86.00±0.20	**86.33±0.17**	83.16±0.33	16.92±0.19	21.45±0.25	3.74±0.01
	3(6612)	**63.95±0.37**	**64.12±0.38**	**63.43±0.63**	14.16±0.38	17.51±0.16	3.81±0.04
	5(10009)	**46.29±0.74**	**46.65±0.87**	**47.26±0.50**	11.61±0.31	13.54±0.32	3.94±0.06
AvgROC	1(2340)	98.35±0.09	**98.77±0.02**	97.89±0.04	58.18±0.04	60.18±0.09	50.04±0.00
	3(6612)	94.31±0.21	**94.63±0.11**	91.57±0.18	57.83±0.13	57.60±0.10	50.04±0.00
	5(10009)	**88.80±0.49**	87.78±0.28	82.77±0.30	56.93±0.12	55.45±0.11	50.04±0.00
1-RankLoss	1(2340)	**99.05±0.04**	98.49±0.03	97.52±0.06	48.17±0.05	33.09±0.04	80.11±0.04
	3(6612)	**96.17±0.09**	94.46±0.10	92.39±0.11	46.31±0.12	28.11±0.19	78.66±0.10
	5(10009)	**92.48±0.26**	90.05±0.23	87.12±0.17	42.54±0.19	22.90±0.17	77.04±0.23
Fmax	1(2340)	**90.10±0.04**	90.02±0.02	76.71±0.18	27.10±0.05	34.21±0.07	25.89±0.01
	3(6612)	**73.60±0.05**	73.15±0.05	61.61±0.21	26.58±0.19	31.29±0.21	25.81±0.03
	5(10009)	**60.44±0.15**	60.28±0.16	53.96±0.32	25.76±0.22	28.28±0.17	25.21±0.09

Table S4 Results of replenishing missing labels on **ScPPI** wrt. GO labels. m is the number of missing labels for a protein and N_m in bracket is the total number of missing labels for all the proteins. The numbers in **boldface** denote the best performance.

Metric	$m(N_m)$	PILL	ProDM	ProWL	LkNN	TPRw	Naive
MicroF1	1(4231)	**95.08±0.05**	93.23±0.02	89.48±0.11	35.32±0.06	37.42±0.01	41.25±0.02
	3(9433)	**88.07±0.08**	84.52±0.05	83.74±0.18	34.92±0.04	36.36±0.04	39.33±0.06
	5(11744)	**83.61±0.15**	80.47±0.02	79.03±0.03	34.96±0.09	35.46±0.03	38.42±0.00
MacroF1	1(4231)	**90.16±0.12**	88.49±0.10	84.31±0.12	23.42±0.12	28.76±0.11	3.70±0.01
	3(9433)	**75.46±0.13**	71.46±0.13	70.25±0.37	21.64±0.09	24.09±0.11	3.53±0.01
	5(11744)	**64.69±0.46**	60.39±0.06	57.15±0.22	20.48±0.07	21.24±0.16	3.46±0.00
AvgROC	1(4231)	**99.07±0.03**	98.69±0.03	98.51±0.03	70.41±0.05	70.41±0.06	43.35±0.00
	3(9433)	**97.23±0.11**	**97.17±0.21**	97.05±0.09	69.15±0.07	66.72±0.10	43.35±0.00
	5(11744)	**94.71±0.20**	94.33±0.42	**94.45±0.25**	68.04±0.14	63.39±0.19	43.35±0.00
1-RankLoss	1(4231)	**99.12±0.14**	98.85±0.02	98.14±0.34	43.54±0.02	40.65±0.03	81.34±0.01
	3(9433)	**97.64±0.12**	96.63±0.30	96.58±0.38	42.78±0.03	38.50±0.06	80.18±0.06
	5(11744)	**96.06±0.16**	93.23±0.32	93.29±0.13	42.09±0.06	36.75±0.05	79.06±0.04
Fmax	1(4231)	**94.07±0.01**	93.95±0.05	77.43±0.05	31.80±0.03	39.90±0.02	38.87±0.00
	3(9433)	**89.59±0.02**	89.47±0.07	74.31±0.12	31.14±0.03	38.82±0.03	37.61±0.00
	5(11744)	87.95±0.01	**88.00±0.01**	72.18±0.12	31.03±0.05	37.82±0.03	37.61±0.00

Table S5 Results of replenishing missing labels on **ScPPI** wrt. FunCat labels. m is the number of missing labels for a protein and N_m in bracket is the total number of missing labels for all the proteins. The numbers in **boldface** denote the best performance.

Metric	$m(N_m)$	PILL	ProDM	ProWL	LkNN	TPRw	Naive
MicroF1	1(4446)	**90.60±0.02**	89.59±0.04	89.40±0.10	28.81±0.06	33.59±0.04	26.88±0.04
	3(12349)	75.11±0.10	73.32±0.06	**76.86±0.19**	27.49±0.13	30.87±0.09	27.17±0.02
	5(18566)	63.82±0.13	61.81±0.15	**65.04±0.23**	26.02±0.18	27.84±0.15	26.62±0.08
MacroF1	1(4446)	85.12±0.22	**85.49±0.17**	84.14±0.24	14.74±0.08	22.64±0.18	2.55±0.01
	3(12349)	60.90±0.20	62.39±0.43	**63.35±0.33**	11.74±0.09	18.61±0.25	2.65±0.01
	5(18566)	43.16±0.48	44.12±0.41	**45.56±0.35**	9.70±0.16	14.59±0.45	2.61±0.02
AvgROC	1(4446)	98.58±0.02	**98.71±0.09**	98.30±0.04	62.41±0.10	66.44±0.07	48.34±0.00
	3(12349)	**94.95±0.18**	**94.94±0.18**	92.91±0.17	62.09±0.15	62.81±0.10	48.34±0.00
	5(18566)	**89.69±0.44**	88.76±0.24	85.07±0.28	61.32±0.16	59.18±0.30	48.34±0.00
1-RankLoss	1(4446)	**99.27±0.01**	99.01±0.02	98.80±0.02	48.98±0.04	36.90±0.03	82.55±0.02
	3(12349)	**96.83±0.10**	96.07±0.05	95.31±0.09	46.96±0.07	31.93±0.16	81.34±0.10
	5(18566)	**93.86±0.12**	92.69±0.12	90.96±0.32	43.67±0.10	26.58±0.24	80.05±0.19
Fmax	1(4446)	**90.12±0.03**	89.90±0.01	78.99±0.29	27.44±0.06	34.46±0.05	23.34±0.00
	3(12349)	**73.26±0.06**	72.95±0.02	63.73±0.32	26.81±0.10	31.83±0.23	23.17±0.08
	5(18566)	60.67±0.26	**61.34±0.06**	60.54±0.20	25.03±0.17	29.06±0.27	22.75±0.04

Table S6 Prediction results on complete unlabeled proteins of *CollinsPPI* wrt. GO labels. The numbers in **boldface** denote the best performance.

Metric	PILL	ProDM	ProWL	LkNN	TPRw	MLR-GL	CIA	Naive
MicroF1	**58.35±0.73**	45.92±1.06	48.55±1.43	44.46±1.21	43.30±1.32	40.35±1.43	44.67±0.81	41.78±1.08
MacroF1	**37.05±1.17**	24.34±1.24	31.53±1.54	34.04±1.16	32.31±1.00	28.43±1.16	31.25±0.57	4.58±0.09
AvgROC	**82.08±0.95**	61.11±0.94	58.03±1.28	66.47±2.12	68.51±1.82	67.22±1.77	63.31±0.51	49.95±1.24
1-RankLoss	**84.19±0.90**	77.19±0.82	65.72±2.35	61.52±1.70	51.48±2.84	48.07±1.87	68.87±1.27	79.06±0.73
Fmax	**66.18±0.83**	25.59±6.96	19.74±1.53	50.24±1.11	57.54±1.57	50.92±1.61	36.47±0.74	44.17±0.72

Table S7 Prediction results on complete unlabeled proteins of **KroganPPI** wrt. GO labels.

Metric	PILL	ProDM	ProWL	LkNN	TPRw	MLR-GL	CIA	Naive
MicroF1	**42.61±0.66**	40.63±0.55	30.74±0.77	28.82±0.82	26.74±0.85	24.53±0.78	27.03±0.95	37.13±0.58
MacroF1	**21.05±0.40**	17.92±0.97	16.36±0.32	17.38±1.27	17.73±0.60	15.84±1.20	15.14±1.27	2.75±0.04
AvgROC	**69.64±0.83**	55.41±0.94	53.61±1.36	61.52±1.13	60.29±1.31	57.89±0.98	57.71±0.77	49.17±0.66
1-RankLoss	75.86±1.06	**79.08±0.58**	48.63±1.25	45.66±1.27	31.76±1.65	37.33±1.39	50.07±2.18	**78.94±0.40**
Fmax	**45.71±1.18**	21.04±8.02	15.73±1.33	34.73±1.29	36.31±1.38	35.70±0.48	26.83±1.08	39.90±0.71

Table S8 Prediction results on complete unlabeled proteins of **KroganPPI** wrt. FunCat labels.

Metric	PILL	ProDM	ProWL	LkNN	TPRw	MLR-GL	CIA	Naive
MicroF1	**29.74±0.69**	27.87±1.20	22.10±0.84	19.40±1.03	20.46±0.30	17.85±0.87	18.74±1.07	23.77±0.40
MacroF1	**13.59±0.74**	11.84±1.15	12.44±0.68	8.75±0.78	11.07±0.47	10.70±1.17	10.26±1.07	1.36±0.02
AvgROC	**61.45±2.05**	55.28±1.16	51.50±1.28	56.85±1.05	56.31±0.93	55.14±1.19	53.18±1.31	49.65±1.13
1-RankLoss	69.90±0.95	**79.66±0.80**	48.81±1.23	30.85±1.68	23.92±0.44	26.31±0.87	46.10±2.01	78.53±0.20
Fmax	**35.70±0.89**	22.15±2.68	13.33±0.68	25.04±1.25	29.38±1.25	29.15±0.83	22.50±0.87	25.54±0.34

Table S9 Experimental results of predicting functions for unlabeled proteins on **ScPPI** wrt. GO labels.

Metric	PILL	ProDM	ProWL	LkNN	TPRw	MLR-GL	CIA	Naive
MicroF1	**39.54±0.52**	27.93±5.89	26.79±0.46	26.45±0.55	26.11±0.75	8.14±0.76	24.92±0.76	35.34±0.20
MacroF1	**14.90±0.45**	11.45±0.76	11.73±0.60	12.56±0.63	**14.43±0.74**	6.54±0.72	12.53±0.83	1.52±0.01
AvgROC	**75.16±0.80**	62.64±0.77	61.40±0.75	67.29±0.58	64.21±0.90	53.79±0.66	63.30±0.60	43.41±0.56
1-RankLoss	77.85±0.85	61.51±9.19	46.87±0.90	44.58±0.67	34.14±1.03	38.35±1.21	49.49±0.99	**80.16±0.37**
Fmax	**43.06±0.68**	25.64±0.62	23.41±7.94	30.89±1.55	36.53±0.77	11.21±2.85	24.13±0.60	37.71±0.09

Table S10 Prediction results on complete unlabeled proteins of **ScPPI** wrt. FunCat labels.

Metric	PILL	ProDM	ProWL	LkNN	TPRw	MLR-GL	CIA	Naive
MicroF1	**30.25±0.52**	21.71±1.43	23.78±0.46	19.84±0.29	22.50±0.64	11.01±0.50	19.12±0.75	21.64±0.12
MacroF1	**11.28±0.53**	**10.43±0.67**	**11.26±0.56**	5.95±0.30	9.82±0.66	7.73±0.61	8.83±0.62	0.85±0.01
AvgROC	**66.95±0.66**	57.87±0.74	56.44±0.78	61.04±0.86	60.29±0.61	55.71±0.73	57.03±1.13	47.74±0.31
1-RankLoss	72.30±1.01	61.27±7.00	50.81±0.68	34.59±0.52	27.98±0.89	38.12±0.95	47.71±0.75	**81.39±0.15**
Fmax	**34.95±0.46**	18.47±9.41	17.87±9.55	26.00±0.88	29.50±0.58	8.58±2.50	21.35±0.53	23.00±0.17

PILL-Jcd on CollingsPPI annotated with FunCat labels. Figs. S3-S5 report the results on CollingsPPI, KroganPPI and and ScPPI with respect to *AvgROC* and *1-RankLoss*. Overall, these results have a trend similar to the results given in the main text.

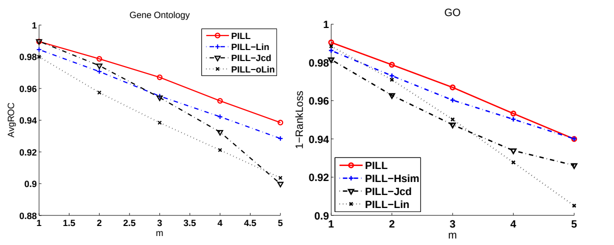

(a) AvgROC (b) 1-RankLoss

Figure S3 The benefit of using function correlation defined by *ComSim*, *HSim*, *LinSim* and Jaccard coefficients on the proteins in *CollingsPPI* annotated with GO labels. PILL uses *ComSim*, PILL-HSim utilizes *Hsim*, PILL-Lin uses *LinSim* and PILL-Jcd is based on Jaccard coefficients.

The benefit of using function label correlation and the guilt by association rule

Figs. S6-S8 give the results on CollingsPPI, KroganPPI, and ScPPI with respect to different metrics (*AvgROC* and *1-RankLoss*). Overall, these results have similar conclusions with the reported results in the main text.

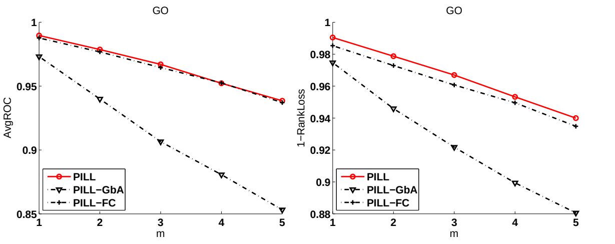

(a) AvgROC (b) 1-RankLoss

Figure S6 The benefit of using function correlation and Guilt by Association rule on the proteins in *CollingsPPI* annotated with GO labels. PILL-FC only uses the function correlation between function labels, PILL-GbA only uses the guilty by association rule, and PILL uses both the function correlation and the guilt by association rule.

We can observe that function correlation alone can replenish the missing labels better than the guilty by association rule alone. It should be noted that without utilizing the guilt by association rule, PILL cannot transfer the labels (including the replenished ones) of the partially labeled proteins to completely unlabeled proteins. Given this, though PILL-FC sometimes performs slightly better than PILL, it's still reasonable to integrate function correlations and the guilt by association rule for protein function prediction.

Author details

References

1. Collins, S.R., Kemmeren, P., Zhao, X.-C., *et al.*: Toward a comprehensive atlas of the physical interactome of saccharomyces cerevisiae. Mol Cell Proteomics **6**(3), 439–450 (2007)
2. Krogan, N.J., Cagney, G., Yu, H., Zhong, G., Guo, X., Ignatchenko, A., Li, J., Pu, S., Datta, N., Tikuisis, A.P., *et al.*: Global landscape of protein complexes in the yeast saccharomyces cerevisiae. Nature **440**(7084), 637–643 (2006)
3. Myers, C.L., Barrett, D.R., Hibbs, M.A., *et al.*: Finding function: evaluation methods for functional genomic data. BMC Genomics **7**(1), 187 (2006)
4. Pandey, G., Myers, C.L., Kumar, V.: Incorporating functional inter-relationships into protein function prediction algorithms. BMC Bioinformatics **10**(1), 142 (2009)
5. Valentini, G.: True path rule hierarchical ensembles for genome-wide gene function prediction. IEEE/ACM Trans Comput Bi **8**(3), 832–847 (2011)
6. Schwikowski, B., Uetz, P., Fields, S.: A network of protein–protein interactions in yeast. Nat Biotechnol **18**(12), 1257–1261 (2000)
7. Zhang, M.-L., Zhou, Z.-H.: A review on multi-label learning algorithms. IEEE Trans Knowl Data En **26**(8), 1819–1837 (2014)
8. Wang, H., Huang, H., Ding, C.: Function–function correlated multi-label protein function prediction over interaction networks. J Comput Biol **20**(4), 322–343 (2013)
9. Yu, G., Domeniconi, C., Rangwala, H., Zhang, G.: Protein function prediction using dependence maximization. In: Proc of European Conf on Machine Learning and Knowledge Discovery in Databases (ECML/PKDD), pp. 574–589 (2013)
10. Yu, G., Rangwala, H., Domeniconi, C., *et al.*: Protein function prediction with incomplete annotations. IEEE/ACM Trans Comput Bi **11**(3), 579–591 (2014)
11. Radivojac, P., Clark, W.T., Oron, T.R., *et al.*: A large-scale evaluation of computational protein function prediction. Nat Methods **10**(3), 221–227 (2013)

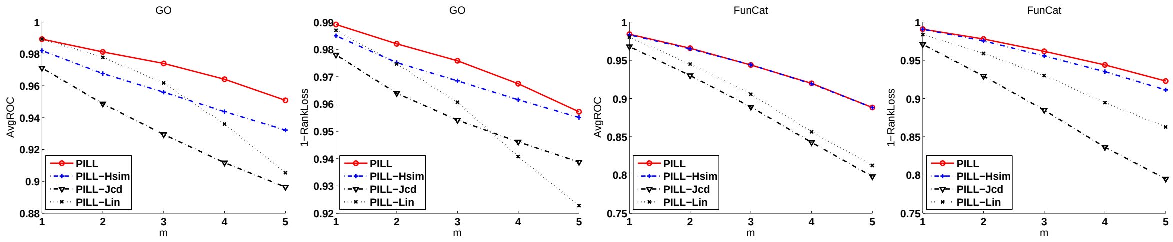

(a) GO (b) GO (c) FunCat (d) FunCat

Figure S4 The benefit of using function correlation defined by *ComSim*, *HSim*, *LinSim* and Jaccard coefficients on the proteins in *KroganPPI* annotated with GO and FunCat labels. PILL uses *ComSim*, PILL-HSim utilizes *Hsim*, PILL-Lin uses *LinSim* and PILL-Jcd is based on Jaccard coefficients.

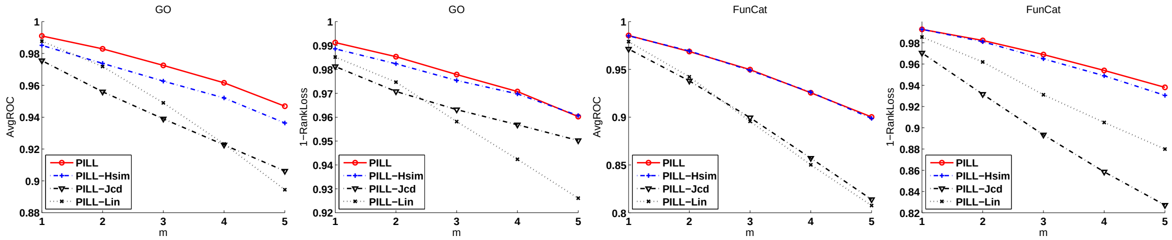

(a) GO (b) GO (c) FunCat (d) FunCat

Figure S5 The benefit of using function correlation defined by *ComSim*, *HSim*, *LinSim* and Jaccard coefficients on the proteins in *ScPPI* annotated with GO and FunCat labels. PILL uses *ComSim*, PILL-HSim utilizes *Hsim*, PILL-Lin uses *LinSim* and PILL-Jcd is based on Jaccard coefficients.

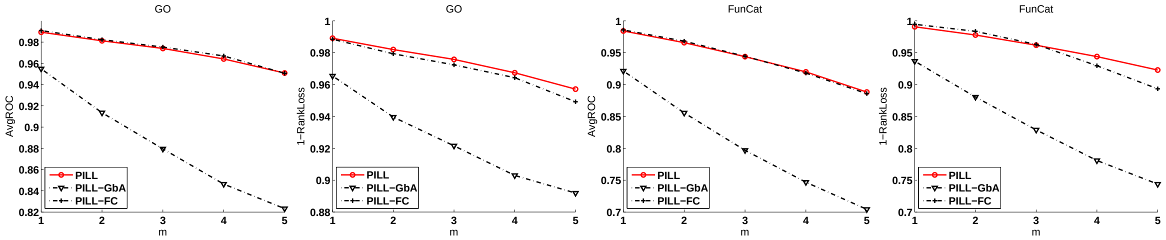

(a) GO (b) GO (c) FunCat (d) FunCat

Figure S7 The benefit of using function correlation and Guilt by Association rule on the proteins in *KroganPPI* annotated with GO and FunCat labels. PILL-FC only uses the function correlation between function labels, PILL-GbA only uses the guilty by association rule, and PILL uses both the function correlation and the guilt by association rule.

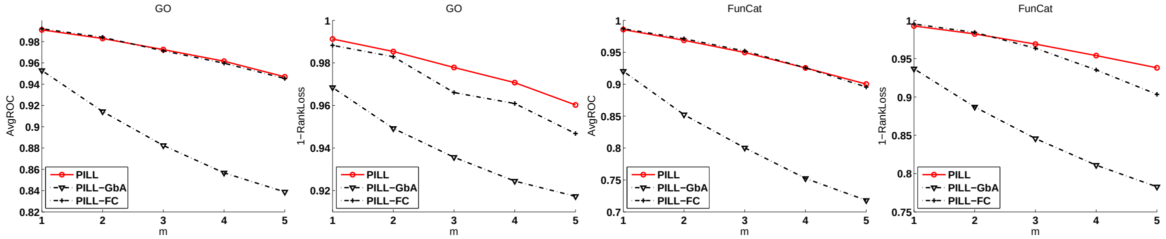

(a) GO (b) GO (c) FunCat (d) FunCat

Figure S8 The benefit of using function correlation and Guilt by Association rule on the proteins in *ScPPI* annotated with GO and FunCat labels. PILL-FC only uses the function correlation between function labels, PILL-GbA only uses the guilty by association rule, and PILL uses both the function correlation and the guilt by association rule.
